# Increased episodes of aspiration on videofluoroscopic swallow study in children with nasogastric tube placement

**DOI:** 10.1371/journal.pone.0227777

**Published:** 2020-01-13

**Authors:** Sarah T. Edwards, Linda Ernst, Ashley K. Sherman, Ann M. Davis

**Affiliations:** 1 Children’s Mercy Kansas City, Division of Gastroenterology, Hepatology and Nutrition, University of Missouri, Kansas City, Missouri, United States of America; 2 Children’s Mercy Kansas City, Division of Hearing and Speech, Kansas City, Missouri, United States of America; 3 Children’s Mercy Kansas City, Health Services and Outcomes Research, Kansas City, Missouri, United States of America; 4 University of Kansas Medical Center, Department of Pediatrics, Kansas City, Kansas, United States of America; 5 Center for Children’s Healthy Lifestyles & Nutrition, Kansas City, Missouri, United States of America; University of Porto Faculty of Medicine, PORTUGAL

## Abstract

**Background:**

Given the limited evidence available, the impact of nasogastric (NG) tube placement on swallowing in children is not well understood. When a child needs to be fed enterally, the current standard is to initially place an NG tube and leave it in place for the first few months of supplemental or total enteral nutrition. It is important to understand if placement of NG tubes has a negative effect on a patient’s swallow.

**Methods:**

We retrospectively reviewed the charts of those children who had videofluoroscopic swallow studies (VFSS) to identify all children who had an NG tube in place at the time of swallow study. Age and sex matched children were identified who had undergone VFSS without an NG in place. These charts were reviewed for diagnosis at the time of the VFSS and presence or absence of aspiration or laryngeal penetrations.

**Results:**

Sixty-three children with NG tubes were identified, along with 63 age and sex matched children without NG tubes in place, at the time of VFSS. Ages ranged from 7 days to 13 years. The NG group had a significantly higher proportion demonstrating aspiration (46% vs. 23.8%, p = 0.0089).

**Conclusions:**

This study supports the need for further prospective evaluation of NG tubes and their effect on swallow, as well as more careful consideration of prolonged NG tube placement in patients with feeding problems. Consideration should be given to removal of the NG prior to VFSS to prevent the impact of NG placement on results of the swallow study which could lead to inappropriate modifications to the patient’s care plan.

## Introduction

Nasogastric tube placement is the typical first step for patients who need supplemental or total enteral nutrition. It is well recognized that nasogastric (NG) tubes may have negative effects on sensory perceptions as well as the potential for oral disorganization [1]. Robbins et al. [2] conducted a study in 80 adults with normal swallow function and demonstrated they had the ability to functionally compensate for the presence of an NG tube without aspirating. When VFSS measurements were compared in adults with and without NG tube placement, those with the tube in place had significantly shorter duration of soft palate elevation (p <0.01), longer duration of maximal hyoid elevation (p <0.01) and anterior excursion (p <0.01), and longer duration of upper esophageal sphincter (UES) opening (p <0.01) (regardless of age or gender group studied). In their discussion, the authors raise the possibility that the presence of an NG tube in dysphagic patients who fatigue easily may cause more than “negligible” effects on the swallow.

Given the lack of evidence otherwise, it is generally accepted among the speech-language pathology community that NG tube placement does not increase aspiration risk. Thus, when a patient has an NG tube in place, VFSS may be performed without the removal of these tubes. However, literature supporting this practice in the pediatric population is sparse. There has been one pediatric study evaluating patients with and without NG tubes, using the patient as their own control, which concluded that there was no significant difference in VFSS findings with and without an NG tube in place [3]. The authors do not specify if the NG tube was in place with the first or second VFSS and noted that the interval between the two studies ranged from 2 weeks to 18 months, with a mode of 3 months [3]. It is possible that any negative swallow function outcomes related to NG tube placement could have been missed by this study due to the confounding variables of maturation and skill development. A significant improvement in swallow function would not be atypical when a VFSS was repeated after an 18 month interval, regardless of NG presence at either time of study.

There are two prospective studies in adult stroke patients assessing swallow changes with and without NG tubes. The swallow was evaluated using fiberoptic endoscopic evaluation of swallowing (FEES) [4–5]. The first of these two studies used aspiration status as their primary outcome and found that there was no statistical difference in aspiration status between those patients with and without NG tubes. However, the latter study [5] did find a significant increase in risk of laryngeal penetration before, during, or after the swallow in patients who had malpositioned NG tubes (i.e., tubes that coiled within the pharynx and/or were more medially displaced with variable contact with the arytenoids). They also identified 2 instances of laryngeal penetration of residual material on the NG tube after the swallow (although there was no significant difference in aspiration risk overall with NG vs. no NG condition) [5]. A third study examined the effect of NG placement on swallow function in adults following stroke using VFSS [6]. This study excluded patients who had more than “minimal” aspiration and noted no significant changes in temporal (oral and pharyngeal transit time) or nontemporal aspects of the swallow with measurements that were compared prior to NG removal and 30 minutes after NG removal. Nontemporal aspects included velopharyngeal closure, pharyngeal contraction, epiglottic tilt, valleculae stasis, pyriform sinus stasis, penetration, and aspiration [6].

When a child needs to be fed enterally the current standard, as recommended by the ASPEN Safe Practices for Enteral Therapy consensus statement, is to select an enteral access device based on the need of the patient [7]. These recommendations give guidelines on which type of enteral access device is appropriate including diagnoses, as well as possible contraindications in specific patients [7]. The recommendation is that NG tubes are for short-term use, typically less than 4–6 weeks [7]. If it is determined that the patient will need supplemental enteral nutrition for a prolonged period of time, placing a gastrostomy tube is recommended. There are times where an NG tube is placed initially to determine patient tolerance prior to moving to a gastrostomy tube. It is especially important to understand whether or not the placement of NG tubes has a negative effect on patient swallow as this may change this routine practice of using an NG tube prior to converting to a gastrostomy tube.

The purpose of the current study was to evaluate the quality of the swallow via VFSS in a sample of children with an NG tube as compared to age and sex matched children without an NG tube. We hypothesized that the presence of an NG tube would decrease swallow function and increase the risk of aspiration.

## Materials and methods

### Study design

Permission for conducting this retrospective study was obtained from the Children’s Mercy Kansas City Institutional Review Board and the requirement of informed consent was waived. We reviewed the charts of those children who had VFSS between January 1, 2011 and December 31, 2011 to identify all children who had an NG tube in place at the time of swallow study. Age and sex matched children were then identified who had undergone VFSS during the same time period without an NG in place. All children, both those with and without NG, underwent VFSS. All speech-language pathologists performing these studies worked exclusively at the same institution and had been trained under the same standardized conditions (with requirement to pass a written and performance-based clinical competency verification assessment). The studies were all performed with the same equipment and objective findings of laryngeal penetrations/aspirations were verified by a radiologist. In cases where there was any ambiguity related to objective findings or interpretation of the studies, the standard protocol dictated that the assessing speech-language pathologist review findings/impressions with one or more speech-language pathologists and/or radiologists until interrater consensus was achieved.

Swallow study reports were reviewed for the presence/absence of aspiration, and presence/absence of laryngeal penetration(s); this information was gathered for each of the ingested consistencies including thin, nectar, thin honey (corresponding to an internal institutional label of “syrup”) and honey consistencies of Varibar^®^ barium. These substances were ranked in order of increasing thickness from 1–4 (with 1 assigned to thin and 4 assigned to honey).

Swallow study reports were also reviewed for diagnoses at the time of swallow study, as recorded by the speech pathologist performing the study. The recorded diagnoses were derived from review of the medical record and parent report at time of patient presentation for the VFSS. The diagnoses were then categorized into 13 different areas including cardiac, ear nose and throat (ENT), failure to thrive (FTT)/malnutrition, gastroenterology, genetic, metabolic/endocrine, neurologic/developmental, oncologic, oral motor, pulmonology, prematurity, renal and rheumatologic. A sample of diagnoses from each of these categories is included (Table 1) but is not exhaustive of the diagnoses represented.

**Table 1:** Diagnostic categories.

Category	Diagnosis
Neuro/Developmental	cerebral agenesis, hydrocephalus, seizure disorder, developmental delay, subdural hemorrhages, hypoxic ischemic encephalopathy
Genetic	Trisomy 21, x-linked opitz G syndrome, Pierre Robin Sequence
GI	GERD, Hirschsprung’es disease
ENT	otitis media, laryngeal cleft, subglottic stenosis, cleft lip, cleft palate, stridor, unilateral vocal cord paralysis
Prematurity	Less than 37 week gestational age
Cardiac	congenital heart disease, ASD, VSD, aortic arch hypoplasia
Metabolic/Endocrine	hypothyroidism
Pulmonary	aspiration, respiratory distress, chronic lung disease, pneumonia, asthma
Rheumatologic	connective tissue disease, arthritis
Oral Motor	dysphagia
FTT/Malnutrition	failure to thrive, malnutrition, weight loss
Renal	renal insufficiency

### Measures

Laryngeal penetration and aspiration events are commonly used outcomes to compare changes in oropharyngeal skills and severity of any related skill deficits noted within videofluoroscopic swallow studies. These outcomes can be used to measure changes within individual patients over time or between groups of participants in research [8]. Both penetration and aspiration events demonstrate compromised airway protection during the swallow, which is a primary function of the pharyngeal swallow phases. Furthermore, there is evidence that deep laryngeal penetrations in pediatric swallow studies are strongly correlated with eventual aspiration during feedings, if feeding is permitted to continue [9]. The viscosity of the liquid or food texture that penetrates or aspirates into the airway typically correlates to the degree of delay in the swallow/airway closure response and overall dysphagia severity [10].

For each patient the “percent aspirated” measurement was calculated by dividing the total number of aspirations by the total number of swallows (“percent aspirated”). The proportion of penetrations and aspirations was calculated by summing the total number of penetrations and aspirations and then dividing by the total number of swallows (“penetrations/aspirations”). We also calculated it for the thickest substance they consumed.

### Statistical analysis

Medians, interquartile ranges (IQR) and proportions were used to summarize the data as the data were not normally distributed. Group differences in categorical variables were analyzed using chi-square or Fisher’s Exact tests. Continuous variables were analyzed using Wilcoxon Rank Sum tests. Spearman correlations were used to look at the relationships between continuous variables. SAS version 9.4 (SAS Institute, Inc. Cary, NC) was used for all analyses. A p-value < 0.05 was considered statistically significant.

## Results

Six hundred and sixty-eight VFSS were conducted during the specified time frame at our site. Of those, 63 children with NG tubes were identified, along with 63 age and sex matched children to serve as controls. Ages ranged from 7 days to 13 years with a median of 0.03 years (IQR: 0.01, 0.04) (Table 2). The sample contained 76 males (60%) and 50 females and the median length of time the NG was in place was 15 days (IQR: 3, 37).

**Table 2:** Patient demographics.

Demographic	NG* (n = 63)	Non − NG* (n = 63)
Gender (%)	50 (39.7) female	50 (39.7) female
Age (range)	7 days–13 years 22 days	8 days–13 years 18 days

*NG nasogastric

The NG and non-NG groups were similar in terms of diagnoses listed in the EMR (Table 3). The only significant difference was that the non-NG group had a higher proportion of gastroenterology patients than the NG group [19 (30%) with NG vs 38 (60%) without NG, p = 0.0007].

**Table 3:** Diagnoses at time of videofluoroscopic swallow study.

Diagnosis	Total n with dx	NG patients with this dx (%) (n = 63)	Non -NG patients with this dx(%) (n = 63)	p-value
Gastroenterology	57	19 (30)	38 (60)	0.0007
Prematurity	45	23 (37)	22 (35)	NS
Pulmonology	43	25 (40)	18 (29)	NS
ENT	33	21 (33)	12 (19)	NS
Cardiac	32	18 (29)	14 (22)	NS
Neurologic/developmental	26	16 (25)	10 (16)	NS
Genetic	21	10 (16)	11 (17)	NS
FTT/malnutrition	15	7 (11)	8 (12)	NS
Oral motor	14	4 (6)	10 (16)	NS
Metabolic/endocrine	5	3 (5)	2 (3)	NS
Renal	5	3 (5)	2 (3)	NS
Oncologic	1	1 (1.59)	0	NS
Rheumatologic	1	0	1 (2)	NS

Note: Children could have multiple diagnoses. Dx = diagnosis; ENT = ear, nose and throat; FTT = failure to thrive; NG = nasogastric; NS = not significant

**Fig 1:**
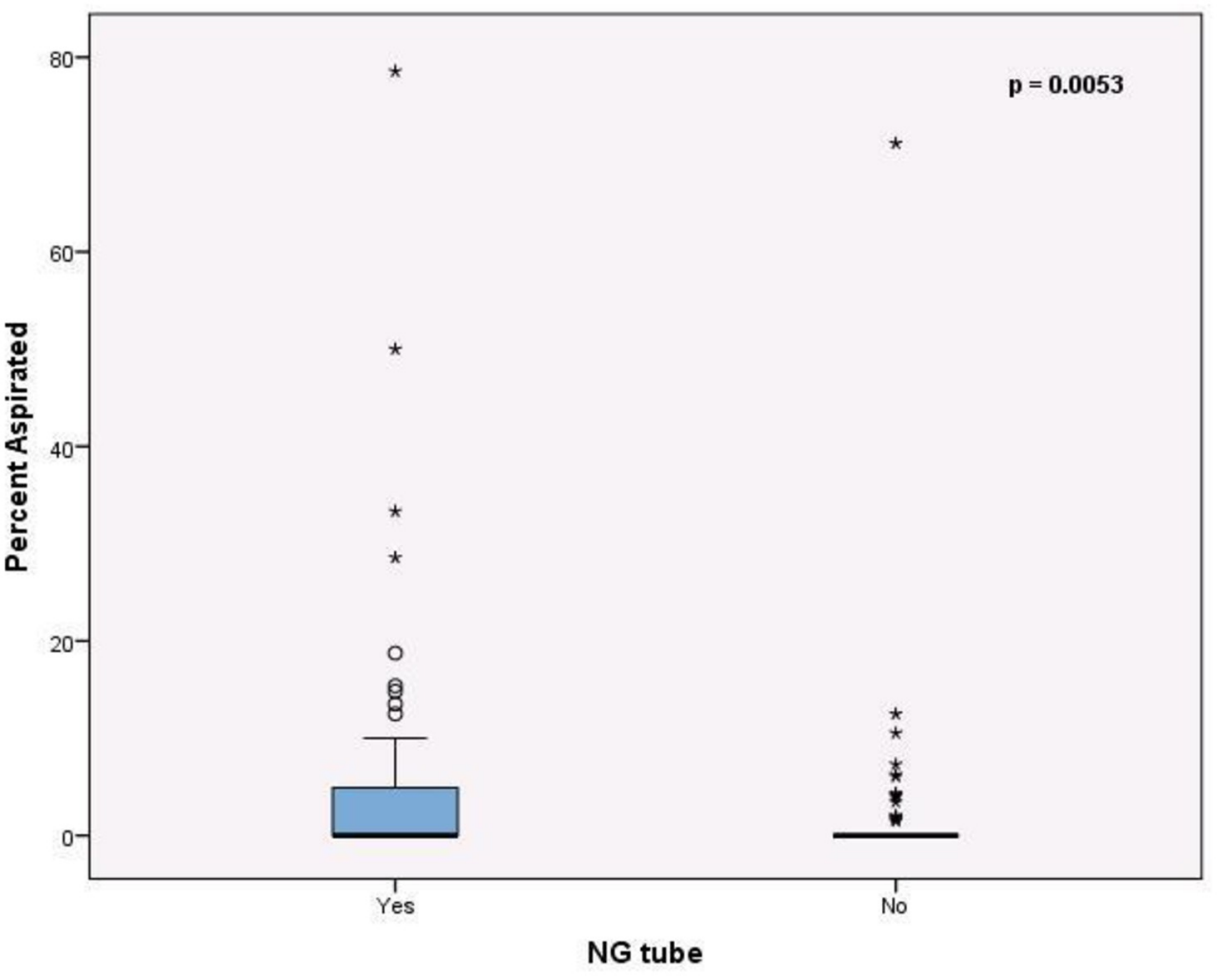
The percent of swallows demonstrating aspiration was significantly different in the presence of an NG tube versus without an NG tube. NG Nasogastric.

In the NG group, the median percent aspirated was 0% (IQR: 0%, 5.2%) and in the non-NG group, the median percent aspirated was 0% (IQR: 0%, 0%); these groups are significantly different (p = 0.0053) [Fig 1]. There were many cases where the percent aspirated was 0, so for each patient the percent aspirated was also dichotomized into no aspiration vs. any aspiration. The NG group had a significantly higher proportion with any aspiration than the non-NG group (29 (46%) vs. 15 (24%), p = 0.0089).

**Fig 2:**
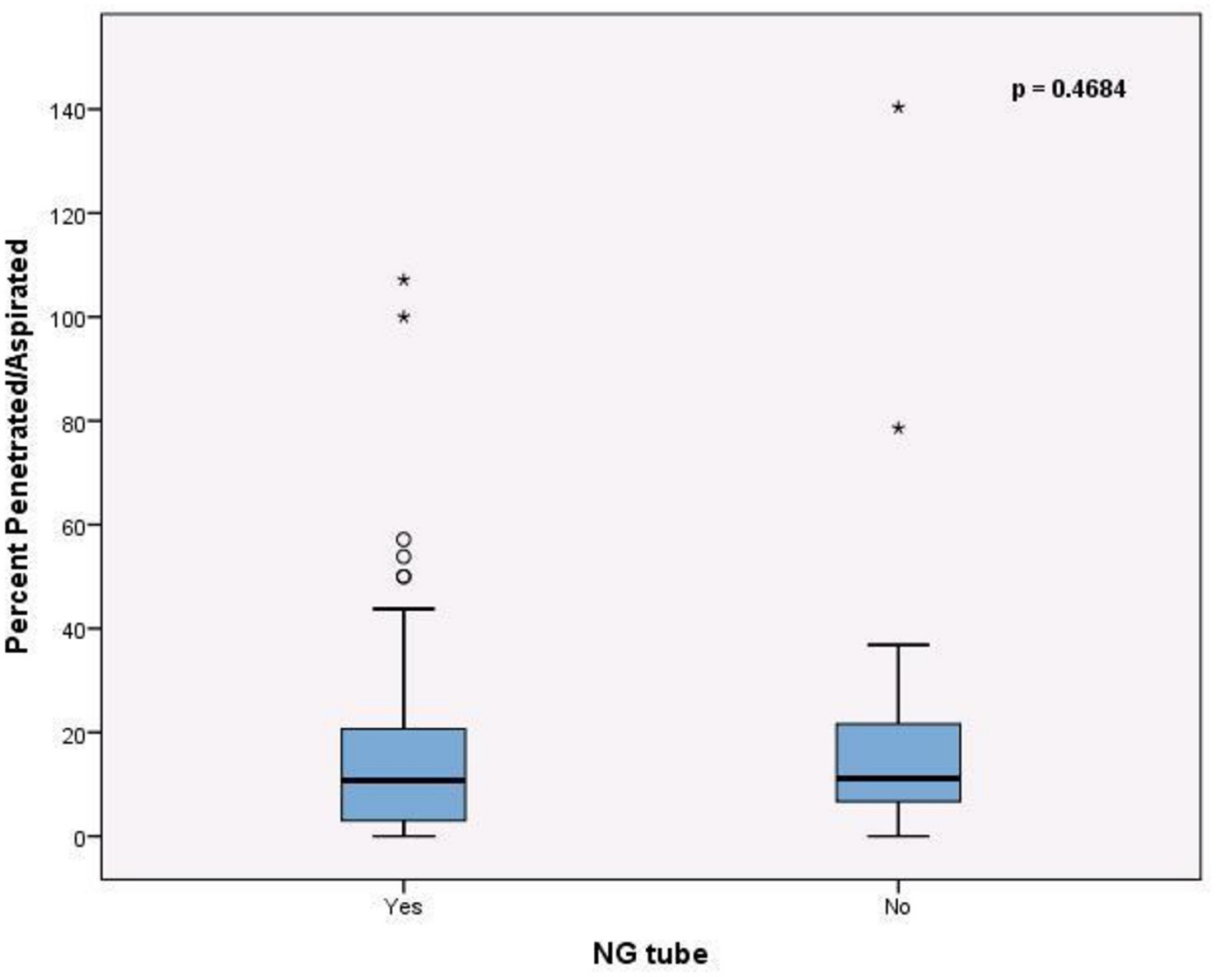
The percent of swallows demonstrating aspiration and penetrations, grouped together was not significantly different between the two groups. NG Nasogastric.

There was no significant difference between the NG group (median 10.7%, IQR: 2.6%, 20.8%) and the non-NG group (median 11.1%, IQR: 5.8%, 22.1%) in terms of the proportion of penetrations/aspirations [Fig 2].

In those with any aspiration in the NG group (n = 53), looking at the thickest consistency on which they aspirated, 13 (24.5%) aspirated on thin, 20 (37.7%) on nectar, 9 (17.0%) on thin honey and 11 (20.8%) on honey. In those with any aspiration in the non-NG group (n = 59), looking at the thickest consistency on which they aspirated, 16 (27.1%) aspirated on thin, 21 (35.6%) on nectar, 11 (18.6%) on thin honey and 11 (18.6%) on honey. These group differences were not statistically significantly different (p = 0.804).

The correlation between the length of time the NG tube was in place and percent aspirated was significant but weak (p = 0.0216, r = -0.289) showing that percent aspirated decreased as the length of time the NG was in place increased. The correlation between duration of NG placement and percent of combined aspiration and penetration events was not statistically significant. However, there was a significant but weak (p = 0.0216, r = -0.289) correlation between the length of time that the NG tube was in place and the percent of aspiration events. Aspiration events decreased as the duration of NG placement increased.

Due to the statistically significant increased number of gastroenterology patients in the non-NG group, we repeated the analyses excluding any gastroenterology patients. The resulting sample consisted of 69 patients, 44 with an NG and 25 without an NG, and there was not a significant difference between the groups in terms of diagnosis category. The NG group maintained a statistically significant higher percentage of aspiration [(NG group median = 1.6%; IQR 0%-6%; non-NG group = 0%, IQR 0%- 0%); p = 0.0158]. The NG group demonstrated a larger proportion with any aspiration [23 (53.3%) vs 6 (24%) p = 0.0222]. No significant difference was demonstrated in the proportion of penetrations/aspirations or difference in the thickest consistency aspirated between the two groups. The correlation between percent aspiration and time the NG was in place was not significant in this sample when gastroenterology patients were removed.

## Discussion

NG tubes are commonly used for initiation of enteral nutrition. Common practices also include placement of an NG tube prior to placement of a gastrostomy tube. The current study sought to better understand whether an NG tube has an effect on the swallow of children. Our results indicate an increase in any aspiration in those patients with an NG tube in place as compared to age and sex matched children without an NG in place. There has been one pediatric study evaluating VFSS in children with and without NG tubes, which found no difference in swallowing events between groups. In this previous study, the patients were their own controls, but the interval between studies with and without NG tube was up to 18 months, which introduces the confounding variables of developmental maturation and skill development related to therapeutic interventions. The current study is the first pediatric study examining aspiration in infants and children with and without NG tubes using age and sex matched controls.

In the current study, diagnoses were comparable between groups with the exception of GI diagnosis, which was increased in those patients without NG placement. GI diagnosis comprised the largest percentage of diagnoses among those reported, with 45% of all patients in the study having a GI diagnosis at the time of swallow study. Fishbein et al. [11], have reported findings of a high incidence of oropharyngeal dysphagia in infants with GERD-like symptoms. Since oropharyngeal dysphagia is commonly associated with gastrointestinal symptoms, the fact that greater frequency of aspiration was not noted in the non-NG group (who had a significant increase in GI symptoms), strengthens support that NG placement rather than underlying medical diagnosis best explains the increased incidence of aspiration in the group of children who had an NG in place at the time of their VFSS. Our results demonstrate that after removing the patients with a gastroenterology diagnosis, a statistically significant increase in any aspiration in the patients with an NG was maintained. This suggests that the statistical significance among patients with gastroenterology diagnoses does not account for the differences in aspiration between the two groups. The other two diagnoses that were not significantly different but which demonstrated a trend (p < 0.05) included the ENT diagnoses (p 0.0682) and the oral motor dysfunction diagnoses (p 0.089). A larger percentage of patients in the NG group has an ENT diagnosis, which include such diagnoses as vocal cord dysfunction, laryngomalacia and cleft palate, all of which could affect risk for aspiration. A larger percentage of patients in the non-NG group had oral motor dysfunction, which could predispose the child to aspiration and could have increased the aspiration rate in the non-NG group. There was not a statistically significant difference with regard to prematurity between the two groups, which is quite important, as there is an increased incidence of oropharyngeal dysphagia in premature infants.

Although healthy adults can adapt their swallow function to accommodate NG tubes [12] children with dysphagia may have impaired ability to do this. Previous authors [2–3] have suggested that individuals with dysphagia may not be able to compensate as well for tube placement by sustaining the upper esophageal sphincter opening longer than normal. This could lead to increased pharyngeal residue, particularly in the pyriform sinuses, which may then aspirate into the airway. This notion is further supported by the common finding that children with dysphagia often have worsening swallow function and increased work of breathing with fatigue. The requirement to compensate for the tube with prolonged hyoid excursion (which also sustains the apneic phase of the swallow via airway closure) and upper esophageal sphincter opening could lead to more rapid deterioration of swallow function.

We did examine the correlation between length time the NG was in place and percent aspiration and found a negative, but significant correlation. This suggests that as the length of time the NG is in place increased, the percent of aspiration decreased. Even though statically significant, it was weakly correlated (r = -0.289). Children who have had an NG tube in place for longer periods of time may have had more time to acclimate to the presence of the NG tube and learn to compensate or may have less impingement on swallow function due to anatomical growth, as well as potential improvement in swallow function with time. It should be noted that in the USA, the ASPEN Safe practices for enteral nutrition [7] indicates that an NG should be placed in situations where enteral nutrition is required for approximately 4–6 weeks and a longer term device in patients needing enteral nutrition for durations exceeding that.

Limitations of this study include the possibility that the patients with NG tubes may have bias for worse swallowing function, by the nature that they need an NG tube when the other group did not. There is also a larger percentage of ENT diagnoses in the NG group, which could possibly account for the differences in aspiration in the two groups. The diagnostic categories were compared but did not delineate those with more severe impairments within each category from those who had more mild impairment. There is potential this could have impacted the results. The bore size and type of NG tube was not collected, so could not be analyzed as an independent variable. It is possible that the relationship between bore size or type of NG tube and age/size of hypopharynx may be an independent variable in aspiration risk. Also, it is not possible to determine if an NG tube is malpositioned during limited lateral view during a VFSS, although an obvious coiling of the tube may have been incidentally identified by the radiologist and was not noted in any report. Due to the fact that some independent data points were not included in some electronic medical record reports, such as the severity of vallecular or pyriform residue after the swallow, it is difficult to isolate the precise factors that led to increased aspiration in the population of children with an NG tube in place. Finally, it is possible that one or more patients assigned to the non-NG cohort had an NG tube that was removed for the purpose of the VFSS which was not reflected in the records reviewed, although this was not a standard practice for conducting VFSS at the time.

More studies are needed to better understand the effects of NG tubes on the pediatric patient. These studies should include not only direct assessment of the tube on the physiology of the swallow (similar to assessment in adults [2]), but also how these effects change over time, the effects NG tubes have on the progression of oral motor skills, and how these change with child age, size, and underlying diagnosis/es. Another important factor regarding the placement of an NG tube not addressed in the current study is parental preference. Considering all of these factors will aid practitioners in decision making when faced with the initiation of supplemental nutrition in their patients.

Our study raises questions regarding the validity of the commonly held belief that NG tubes do not affect aspiration risk in children. Consideration should be given to removal of the NG prior to VFSS to prevent the impact of NG placement on results of the swallow study which could lead to inappropriate modifications to the patient’s care plan.

This study supports the need for further prospective evaluation of NG tubes on the pediatric swallow, as well as more careful consideration of prolonged NG tube placement. Results also suggest that caution should be used in the interpretation of a swallow study performed in the presence of an NG tube. Although there is some evidence for a need to change practice, future studies hope to further delineate circumstances under which the NG tube should be removed.

## Supporting information

S1 Dataset This is a file composed of the raw data. (XLSX)
